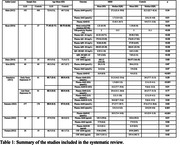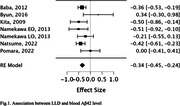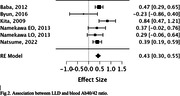# Association Between Late‐Life Depression and Amyloid Beta Burden in Older Adults Without Cognitive Impairment: A. Systematic Review and Meta‐Analysis

**DOI:** 10.1002/alz.095618

**Published:** 2025-01-09

**Authors:** Natalia Pozo Castro, Regina Silva Paradela, Isabel Elaine Allen, Karla Paulina Ruiz‐Castillo, Agathe Vrillon, Carolina Delgado, Charles Windon

**Affiliations:** ^1^ Hospital San Borja Arriarán, Santiago, Region Metropolitana Chile; ^2^ Global Brain Health Institute, Memory and Aging Center, University of California San Francisco, San Francisco, CA USA; ^3^ University of Chile, Santiago, Region Matropolitana Chile; ^4^ University of São Paulo Medical School, São Paulo, São Paulo Brazil; ^5^ Department of Epidemiology and Biostatistics, University of California, San Francisco, San Francisco, CA USA; ^6^ Global Brain Health Institute, University of California San Francisco, San Francisco, CA USA; ^7^ Memory and Aging Center, UCSF Weill Institute for Neurosciences, University of California, San Francisco, San Francisco, CA USA; ^8^ University of California San Francisco, San Francisco, CA USA; ^9^ Global Brain Health Institute, San Francisco, CA USA; ^10^ National Institute of Neurology and Neurosurgery Manuel Velasco Suarez / Mexican Social Security Institute, Torreón, CU Mexico; ^11^ Memory and Aging center, UCSF, San Francisco, CA USA; ^12^ Universidad de Chile, Santiago, Region Metropolitana Chile; ^13^ Hospital Clínico Universidad de Chile, Santiago, Region Metropolitana Chile; ^14^ Memory and Aging Center, Weill Institute for Neurosciences, University of California, San Francisco, San Francisco, CA USA; ^15^ Department of Neurology, University of California, San Francisco, San Francisco, CA USA

## Abstract

**Background:**

Late‐life depression (LLD) is a risk factor for cognitive impairment. Some studies have shown an association between higher amyloid‐beta (Aβ) burden, a known marker of Alzheimer’s Disease (AD), and LLD, but research findings are mixed. We performed a systematic review and meta‐analysis to compare the Aβ burden between cognitively unimpaired subjects with and without LLD.

**Method:**

We searched MEDLINE, Embase, and ScienceDirect for studies comparing Aβ burden between LLD patients and controls (molecular imaging and/or fluid‐biomarker confirmation) and included subjects aged ≥60 years with LLD, and absence of cognitive impairment (MMSE ≥24 and/or description of normal cognition). The review process was performed by 2 independent reviewers (Covidence‐software). A random effects meta‐analysis was executed to evaluate the pooled difference of Aβ burden between LLD and control groups (JASP‐software).

**Result:**

372 studies were identified and eight met the inclusion‐exclusion criteria (combined sample: 725 LLD subjects and 885 controls). The weighted‐mean age was 69.3 and 68.9 years for subjects and controls. Six studies evaluated Aβ in blood (4 on serum and 2 on plasma, one of them also evaluated PET‐Aβ), and two evaluated cerebrospinal fluid (CSF) (table 1). One of the serum studies (Namekawa) included two subgroups, one with subjects that besides depression after 60 years old also had a history of depression before age 60, and one with participants with a first episode of major depressive disorder after 60 years old. Both subgroups were analyzed separately in the meta‐analysis. The meta‐analysis of plasma and serum studies showed that individuals with LLD had lower levels of Aβ42 (estimate ‐0.344, p<0.001) (Fig. 1) and higher levels of Aβ40/42 ratio (estimate 0.428, p<0.001) compared to controls (Fig. 2). The results remained significant when plasma and serum were analyzed independently.

**Conclusion:**

In this systematic review and meta‐analysis, we found significantly lower levels of Aβ42 and higher levels of Aβ40/42 ratio in cognitively unimpaired older adults with LLD compared to controls. These findings support the idea that LLD is associated with AD pathology. This is a relevant area of research for increasing the understanding of AD’s clinical presentation that could potentially impact access to early diagnosis and treatment.